# TRAF2 inhibits TRAIL- and CD95L-induced apoptosis and necroptosis

**DOI:** 10.1038/cddis.2014.404

**Published:** 2014-10-09

**Authors:** I Karl, M Jossberger-Werner, N Schmidt, S Horn, M Goebeler, M Leverkus, H Wajant, T Giner

**Affiliations:** 1 Department of Dermatology, Venereology and Allergology, University Hospital Würzburg, Würzburg, Germany; 2 Department of Dermatology, Venereology and Allergology, University Medical Center Mannheim, University of Heidelberg, Mannheim, Germany; 3 Division of Molecular Internal Medicine, Department of Internal Medicine II, University Hospital Würzburg, Würzburg, Germany

## Abstract

The relevance of the adaptor protein TNF receptor-associated factor 2 (TRAF2) for signal transduction of the death receptor tumour necrosis factor receptor1 (TNFR1) is well-established. The role of TRAF2 for signalling by CD95 and the TNF-related apoptosis inducing ligand (TRAIL) DRs, however, is only poorly understood. Here, we observed that knockdown (KD) of TRAF2 sensitised keratinocytes for TRAIL- and CD95L-induced apoptosis. Interestingly, while cell death was fully blocked by the pan-caspase inhibitor benzyloxycarbonyl-Val-Ala-Asp(OMe)-fluoromethylketone (zVAD-fmk) in control cells, TRAF2-depleted keratinocytes were only partly rescued from TRAIL- and CD95L-induced cell death. In line with the idea the only partially protective effect of zVAD-fmk on TRAIL- and CD95L-treated TRAF2-depleted keratinocytes is due to the induction of necroptosis, combined treatment with zVAD-fmk and the receptor interacting protein 1 (RIP1) inhibitor necrostatin-1 that fully rescued these cells. To better understand the impact of TRAF2 levels on RIP1- and RIP3-dependent necroptosis and RIP3-independent apoptosis, we performed experiments in HeLa cells that lack endogenous RIP3 and HeLa cells stably transfected with RIP3. HeLa cells, in which necroptosis has no role, were markedly sensitised to TRAIL-induced caspase-dependent apoptosis by TRAF2 KD. In RIP3-expressing HeLa transfectants, however, KD of TRAF2 also strongly sensitised for TRAIL-induced necroptosis. Noteworthy, priming of keratinocytes with soluble TWEAK, which depletes the cytosolic pool of TRAF2-containing protein complexes, resulted in strong sensitisation for TRAIL-induced necroptosis but had only a very limited effect on TRAIL-induced apoptosis. The necroptotic TRAIL response was not dependent on endogenously produced TNF and TNFR signalling, since blocking TNF by TNFR2-Fc or anti-TNF*α* had no effect on necroptosis induction. Taken together, we identified TRAF2 not only as a negative regulator of DR-induced apoptosis but in particular also as an antagonist of TRAIL- and CD95L-induced necroptosis.

Death receptors (DRs) constitute a subgroup of the tumour necrosis factor receptor (TNFR) superfamily and are characterised by their cytoplasmic ‘death domain'. Stimulation of the prototypic DRs TNF-related apoptosis inducing ligand (TRAIL) receptor-1 (TRAILR1), TRAILR2 or CD95 by their ligands TRAIL and CD95L leads to recruitment of the adaptor proteins Fas-associated death domain (FADD) and of caspase-8. In the so formed death inducing signalling complex (DISC), caspase-8 undergoes proximity-induced maturation to the enzymatically fully active heterotetrameric caspase-8 molecule which is released from the DISC.^1,2^ TNFR1 is also a DR, but acts *in vivo* primarily as a driver of inflammatory processes and less as an inducer of cell death. TNFR1 signals apoptosis not via a receptor-associated DISC but via a secondarily formed cytosolic caspase-8 activating complex.^3,4^

Keratinocytes express TRAILR1 and TRAILR2 as well as CD95 and can undergo apoptosis upon TRAIL or CD95L stimulation under defined circumstances.^5, 6, 7^ The well-balanced activity of DR-associated apoptotic and non-apoptotic signalling pathways in the skin is thus crucial for skin physiology. Pathological disturbance of the balance between these pathways may lead to skin cancer or inflammatory skin conditions such as psoriasis, alopecia areata or toxic epidermal necrolysis.^8,9^

Noteworthy, DRs may also trigger a caspase-independent mode of cell death.^10,11^ This caspase-independent form of DR-induced programmed cell death has been termed necroptosis and is characterised by swelling of the organelles, increased cell volume and disruption of the plasma membrane subsequently leading to inflammation.^12,13^ Mechanisms that inhibit necroptosis are critical for maintenance of tissue homeostasis. So far, induction of necroptosis has mainly been investigated after stimulation of TNFR1 or CD95, but has also been suggested for TRAIL.^14, 15, 16^

At the molecular level one crucial determinant of the quality of the DR response is the serine threonine kinase RIP1. Like FADD and caspase-8, RIP1 is recruited to liganded DRs but it is also part of cytosolic caspase-8 activating complexes formed in response to TNF, genotoxic stress or depletion of the E3 ligases cellular inhibitor of apoptosis protein 1 and 2 (cIAP1 and cIAP2).^4,17, 18, 19, 20^ Dependent on the availability of the anti-apoptotic caspase-8 homologue FLICE-inhibitory protein long (FLIP-L), cIAP1/2 and the RIP1-related kinase RIP3, the cytoplasmic RIP1/caspase-8 containing complexes can lead to RIP3-dependent necroptosis^12^ or caspase-8-mediated apoptosis,^4^ but presumably also to RIP1-mediated activation of the anti-apoptotic classical nuclear factor *κ*B (NF*κ*B) pathway.^21,22^ The kinase activity of RIP1 is required for the induction of necroptosis but seems to be dispensable for its NF*κ*B and caspase-8 stimulating activities.^23,24^

TRAF2 has been initially described as a cytosolic adaptor protein that not only interacts with the cytoplasmic part of various non-DRs of the TNFR family but also mediates recruitment of the E3 ligases cIAP1 and cIAP2.^25^ It turned out that the TRAF2/cIAP complex is also indirectly recruited to some DRs, particularly to TNFR1.^26^ TRAF2 and the cIAPs typically contribute to a varying extent to stimulation of signalling pathways, resulting in the activation of NF*κ*B transcription factors and MAP kinases. While the K63 E3 ligase activity of cIAP1 and cIAP2 is of pivotal relevance there is also evidence that TRAF2 can act itself as a K48 E3 ligase of caspase-8 that triggers proteasomal degradation of the maturated enzyme.^27^ However, the E3 ligase activity of TRAF2 is controversially discussed.^28, 29, 30^

In the current study, we investigated the relevance of TRAF2 for the cell death response to TRAIL and CD95L stimulation. It turned out that TRAF2 is involved in protection against TRAIL- and CD95L-induced apoptosis but above all against TRAIL- and CD95L-induced necroptosis.

## Results

### TRAF2 knockdown sensitises HaCaT keratinocytes for TRAIL-induced cell death

**Figure 1:**
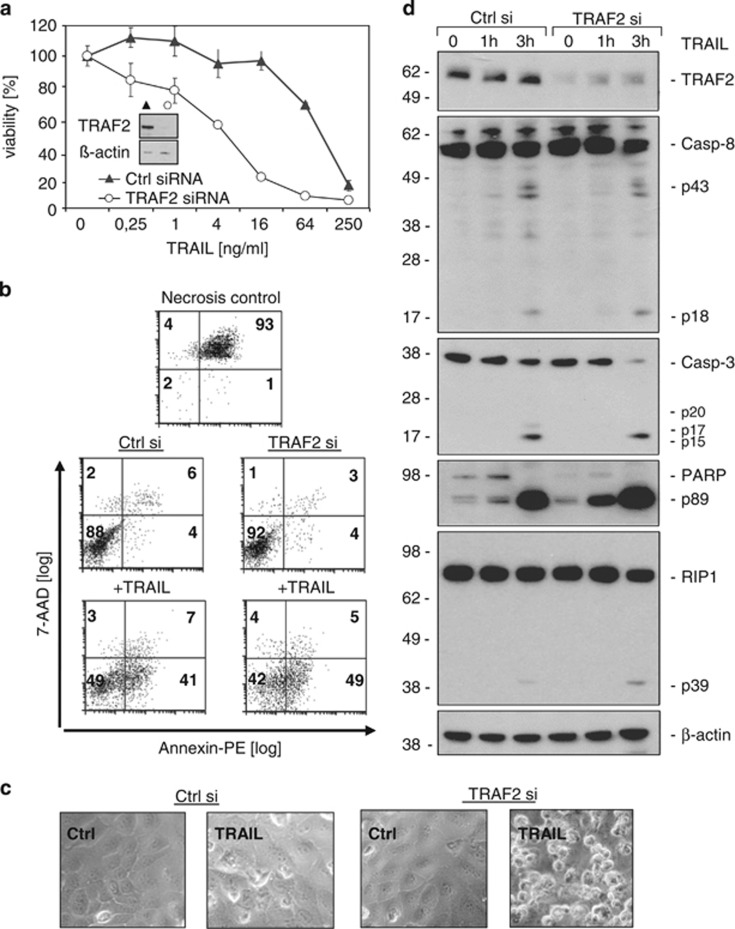
TRAF2 knockdown sensitises HaCaT keratinocytes for TRAIL-induced cell death. HaCaT keratinocytes were transfected with control siRNA and TRAF2 siRNA. Experiments were performed 24–96 h after transfection. Knockdown efficiency was controlled by Western blotting (insert in a). (**a**) HaCaT cells treated with control siRNA or TRAF2 siRNA were stimulated in triplicates with the indicated concentrations of Killer-TRAIL for 18 h and viability was measured by crystal violet staining. One representative experiment of four independent experiments is shown. Heat-shocked (55 °C for 30 min) cells were analysed as a positive control for the detection of necrotic cells. (**b**) HaCaT cells transfected with control siRNA and TRAF2 siRNA were stimulated for 6 h with Killer-TRAIL (64 ng/ml), stained with Annexin-PE and 7-AAD and finally analysed by flow cytometry. (**c**) HaCaT keratinocytes with or without TRAF2 knockdown were stimulated with 4 ng/ml Killer-TRAIL for 18 h and cellular morphology was documented by light microscopy. (**d**) Cells transfected with control and TRAF2-targeting siRNA were stimulated with 64 ng/ml Killer-TRAIL. After 1 and 3 h total cell lysates were prepared and subjected to Western Blot analysis to detect the indicated proteins

To investigate the relevance of TRAF2 for TRAIL DR-induced cell death in keratinocytes, TRAF2 was depleted in HaCaT cells by RNA interference. Cells were then stimulated with Killer-TRAIL, a highly active, commercially available form of TRAIL, and evaluated for viability and molecular markers of cell death. HaCaT keratinocytes with reduced TRAF2 levels were markedly sensitised for TRAIL-induced cell death. Crystal violet staining revealed an ~16-fold lower lethal dose 50% for Killer-TRAIL-induced cell death in TRAF2 small interfering RNA (siRNA)-transfected cells as compared with cells transfected with an irrelevant control siRNA (Figure 1a). In line with this finding, TRAIL-induced externalisation of phosphatidylserine was increased in TRAF2 knockdown (KD) cells (Figure 1b) and came along with increased detachment from the plastic surface (Figure 1c). TRAIL-induced cell death was furthermore accompanied by activation and cleavage of caspases and this was also moderately enhanced after TRAF2 KD. Cleavage of caspase-8 and caspase-3 as well as of the caspase-3 substrate PARP and of RIP1 was equal or even more intense in the TRAF2 KD keratinocytes after 1–3 h (Figure 1d).

### TRAIL induces necroptosis in HaCaT keratinocytes upon TRAF2 KD and caspase inhibition

**Figure 2:**
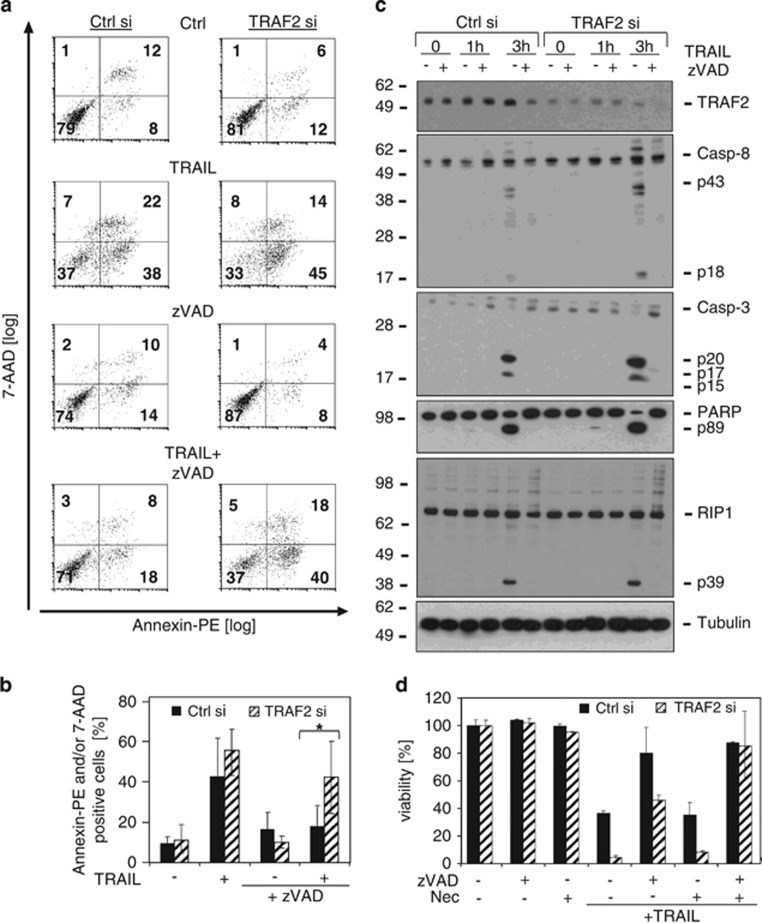
TRAIL induces necroptosis in HaCaT keratinocytes upon TRAF2 knockdown and caspase inhibition. HaCaT keratinocytes were transfected with control siRNA or TRAF2 siRNA. Experiments were performed 24–96 h after transfection. (**a**) Control siRNA and TRAF2 siRNA-transfected HaCaT cells were pretreated with the pan-caspase inhibitor zVAD-fmk (40 *μ*M, 30 min) and then stimulated with Killer-TRAIL (64 ng/ml) for 8 h. Finally, cells were stained with Annexin-PE and 7-AAD and subjected to flow cytometric analysis. A diagram depicting the mean values of all three experiments is shown in **b**. Statistically significant differences (*P*≤0,05; student's *t*-test) were marked by *. (**c**) siRNA transfected HaCaT keratinocytes were treated with Killer-TRAIL (100 ng/ml) for 1 h or 3 h with or without zVAD-fmk. Western blot analysis was performed to detect the indicated proteins. (**d**) Control siRNA or TRAF2 siRNA-transfected HaCaT cells were pretreated with the indicated mixtures of the pan-caspase inhibitor zVAD-fmk (40 *μ*M) and the RIP1 inhibitor necrostatin-1 (90 *μ*M) for 60 min and then cells were challenged with Killer-TRAIL (16 ng/ml). Cell viability was quantified by crystal violet staining after 20 h

TRAIL-induced cell death correlated with activation of caspases. To explore whether caspase activation is required for TRAIL-induced cell death in TRAF2 KD cells or whether necroptosis contributed to overall cell death, we next analysed the effects of the pan-caspase inhibitor zVAD-fmk and of the RIP1-inhibitor necrostatin-1 (Nec) on TRAIL-induced cell death. In cells transfected with control siRNA, TRAIL-induced cell death was largely blocked by zVAD-fmk (Figures 2a and b). Surprisingly, TRAF2 KD not only sensitised for TRAIL-induced cell death, but also changed the character of the TRAIL cell death response. In the presence of zVAD-fmk, and in marked contrast to control cells, TRAF2 siRNA-transfected cells were only partly rescued by zVAD-fmk (Figures 2a and b). This could also be reproduced with independent functional siRNA's for TRAF2 (Supplementary Figure S8). To confirm that caspase inhibition by zVAD-fmk was effective, we performed Western blot analysis of TRAIL-exposed TRAF2-depleted and control siRNA-transfected HaCaT cells. Treatment with zVAD-fmk indeed prevented TRAIL-induced processing of caspases-3 and -8 in control siRNA- and TRAF2 siRNA-transfected cells (Figure 2c). Preincubation with the RIP1 inhibitor necrostatin-1 alone showed no protective effect on TRAIL-induced cell viability irrespective of TRAF2 KD (Figure 2d). However, in contrast to zVAD-fmk, a mixture of zVAD-fmk and necrostatin-1 completely rescued TRAIL-stimulated TRAF2-depleted keratinocytes (Figure 2d). These data indicate that TRAF2 KD sensitises keratinocytes for RIP1-mediated necroptosis under conditions of impaired caspase-8 activation.

### CD95 stimulation leads to increased apoptosis and necroptosis in TRAF2 KD HaCaT keratinocytes

**Figure 3:**
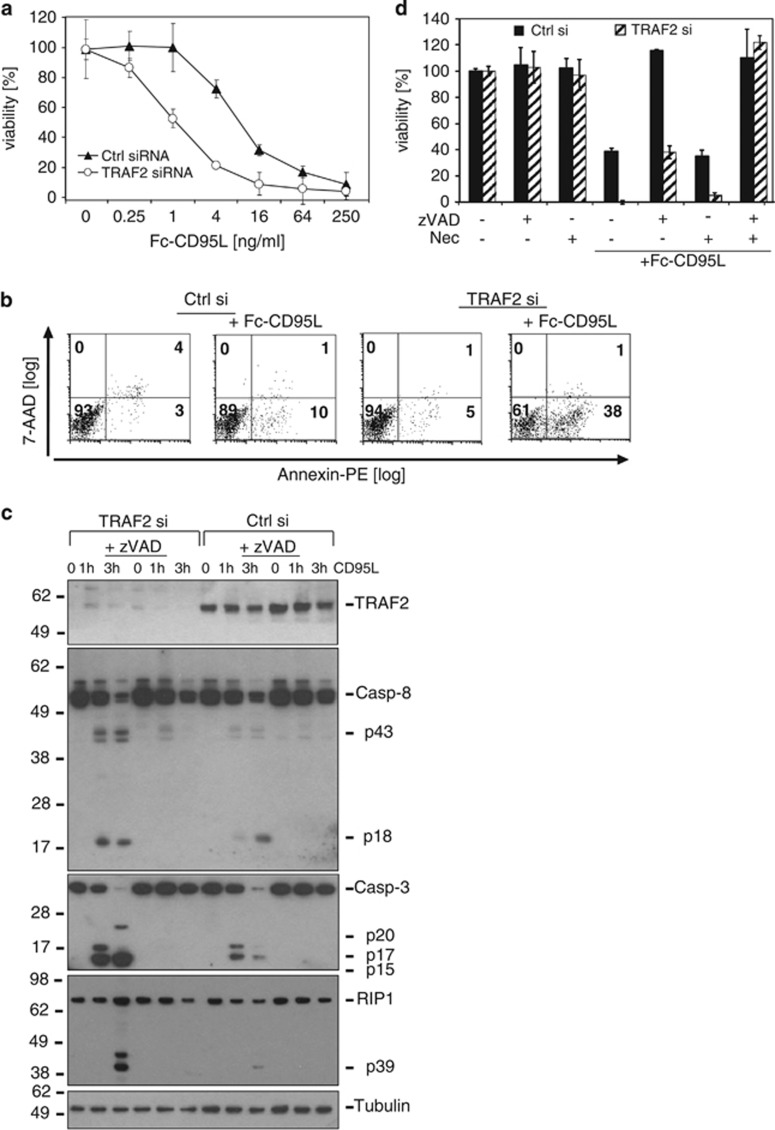
Induction of apoptosis and necroptosis by Fc-CD95L is enhanced in TRAF2 knockdown HaCaT keratinocytes. Experiments were performed essentially as described in the legends of Figures 1 and 2 but cells were stimulated with Fc-CD95L instead of TRAIL. (**a**) Control siRNA- and TRAF2 siRNA-transfected HaCaT cells were stimulated overnight with Fc-CD95L and finally analysed for their viability using crystal violet staining. (**b**) Cells were stimulated with Fc-CD95L (64 ng/ml) for 6 h and then stained with Annexin-PE and with 7-AAD. The numbers in each quadrant indicate the percentage of all cells present in the corresponding quadrant. (**c**) TRAF2 siRNA and control siRNA-transfected HaCaT were pretreated with DMSO (solvent control) or zVAD-fmk (40 *μ*M) for 30 min and were then stimulated with Fc-CD95L (250 ng/ml) for 1 or 3 h. Total cell lysates were analysed by Western blotting for the presence of the indicated proteins. One out of two representative experiments is shown. (**d**) HaCaT cells were treated with the indicated siRNAs. The next day, cells were preincubated with zVAD-fmk (40 *μ*M) alone or in combination with necrostatin-1 (90 *μ*M) for 60 min and then stimulated with Fc-CD95L (16 ng/ml) overnight. Viability was measured by crystal violet staining

CD95 and the TRAIL DRs signal by quite similar mechanisms. Therefore, we next analysed CD95-mediated cell death in TRAF2 KD keratinocytes. CD95L-treated HaCaT cells with TRAF2 KD exhibited a higher sensitivity to CD95L-induced cell death as compared with controls (Figures 3a and b). Caspase-8 and caspase-3 processing as well as cleavage of RIP1 was enhanced in TRAF2 KD HaCaT cells and was fully inhibited by preincubation with zVAD-fmk (Figure 3c). CD95-mediated cell death, however, was fully blocked by caspase inhibition only in the control siRNA-transfected, but not in the TRAF2 siRNA-transfected HaCaT cells (Figure 3d). As with TRAIL, the caspase-independent form of cell death occurring in CD95-stimulated zVAD-fmk-treated TRAF2 KD cells was rescued in the presence of necrostatin-1 (Figure 3d).

### TRAF2 KD in primary keratinocytes unleashes DR-induced necroptosis when caspase activity is impaired

**Figure 4:**
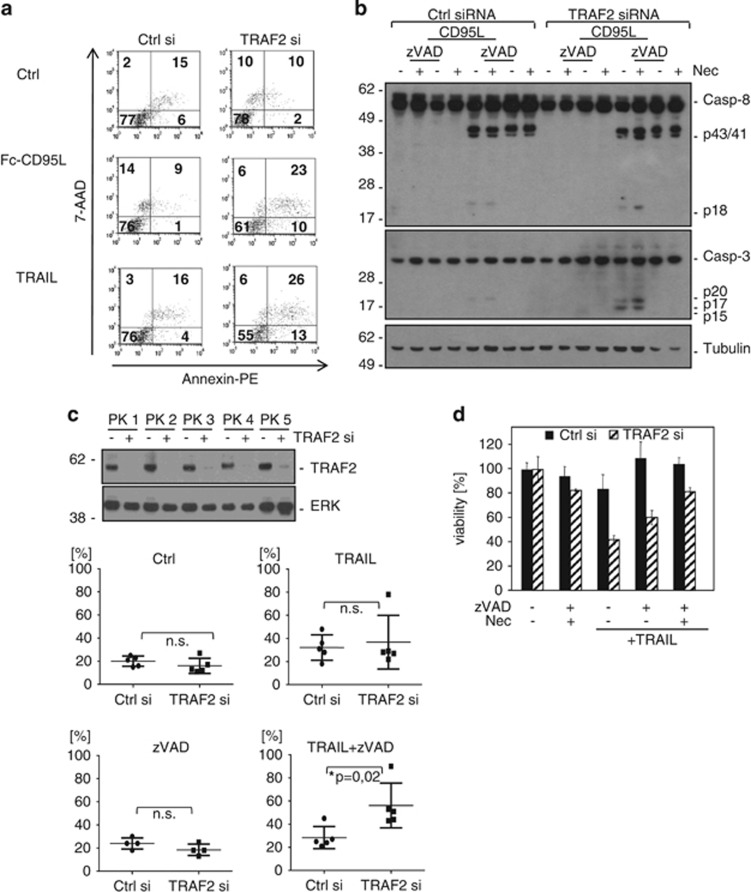
TRAF2 knockdown in primary keratinocytes unleashes death receptor-induced necroptosis when caspase activity is impaired. (**a**) Primary human keratinocytes were transiently transfected with TRAF2 siRNA or control siRNA. 24 h after transfection cells were stimulated with Fc-CD95L (64 ng/ml) or TRAIL (64 ng/ml). After 6 h, cells were stained with Annexin-PE and 7-AAD and evaluated by flow cytometric analysis. (**b**) Two days after siRNA transfection, primary keratinocytes were preincubated for 1 h with zVAD-fmk (40 *μ*M) or necrostatin-1 (90 *μ*M), either alone or in combination. Cells were then stimulated with Fc-CD95L (64 ng/ml) for additional 3 h and processing of caspase-8 and caspase-3 was analysed by Western blotting. Tubulin served as a load control. (**c**) Primary keratinocytes obtained from five different donors (PK1-PK5) were transfected either with TRAF2 siRNA or control siRNA (transfection efficiency is shown in the upper panel). Transfected primary keratinocytes were preincubated with zVAD-fmk (40 *μ*M) and then stimulated with Killer-TRAIL (64 ng/ml) for 6 h. Cells were then stained with Annexin-PE and 7-AAD and analysed by flow cytometry. The diagrams depicted in the lower panel show the percentage of Annexin-PE and/or 7-AAD-positive keratinocytes from all five donors at the indicated conditions. Statistically significant differences (Student's *t*-test) are indicated. (**d**) Primary keratinocytes were transfected with the indicated siRNAs. The next day, cells were preincubated with zVAD-fmk (40 *μ*M) alone or in combination with necrostatin-1 (90 *μ*M) for 60 min. Cells were then stimulated with TRAIL (100 ng/ml) overnight and viability was measured by crystal violet staining. Experiments revealed similar results in keratinocytes from three different donors

HaCaT cells are a spontaneously transformed keratinocyte cell line that shares many but not all properties of primary keratinocytes.^31^ We therefore analysed the effects of TRAF2 KD on TRAIL- and CD95L-induced cell death in primary keratinocytes. In contrast to HaCaT cells, primary keratinocytes express high levels of FLIP-L and are therefore largely resistant towards TRAIL- and CD95L-induced cell death.^6^ Interestingly, KD of TRAF2 enabled CD95L and TRAIL to induce cell death in primary keratinocytes (Figures 4a and d). Western Blot analysis furthermore revealed increased caspase-3 processing in TRAF2 KD keratinocytes after CD95L stimulation, whereas caspase-8 cleavage to p43/41 remained largely unchanged (Figure 4b). Intriguingly, preincubation with zVAD-fmk inhibited DR-induced processing of caspase-8 and caspase-3 to p18 and p20/17/15 but enhanced cell death induction in the TRAF2 KD cells (Figures 4b and c). Co-incubation with necrostatin-1 and zVAD-fmk restored TRAIL resistance in control-transfected as well as in TRAF2 KD keratinocytes (Figure 4d). This argues for an anti-necroptotic activity of TRAF2 in the context of DR signalling not only in HaCaT cells but also in primary keratinocytes.

### RIP3 expression confers competence for TRAIL-induced necroptosis

**Figure 5:**
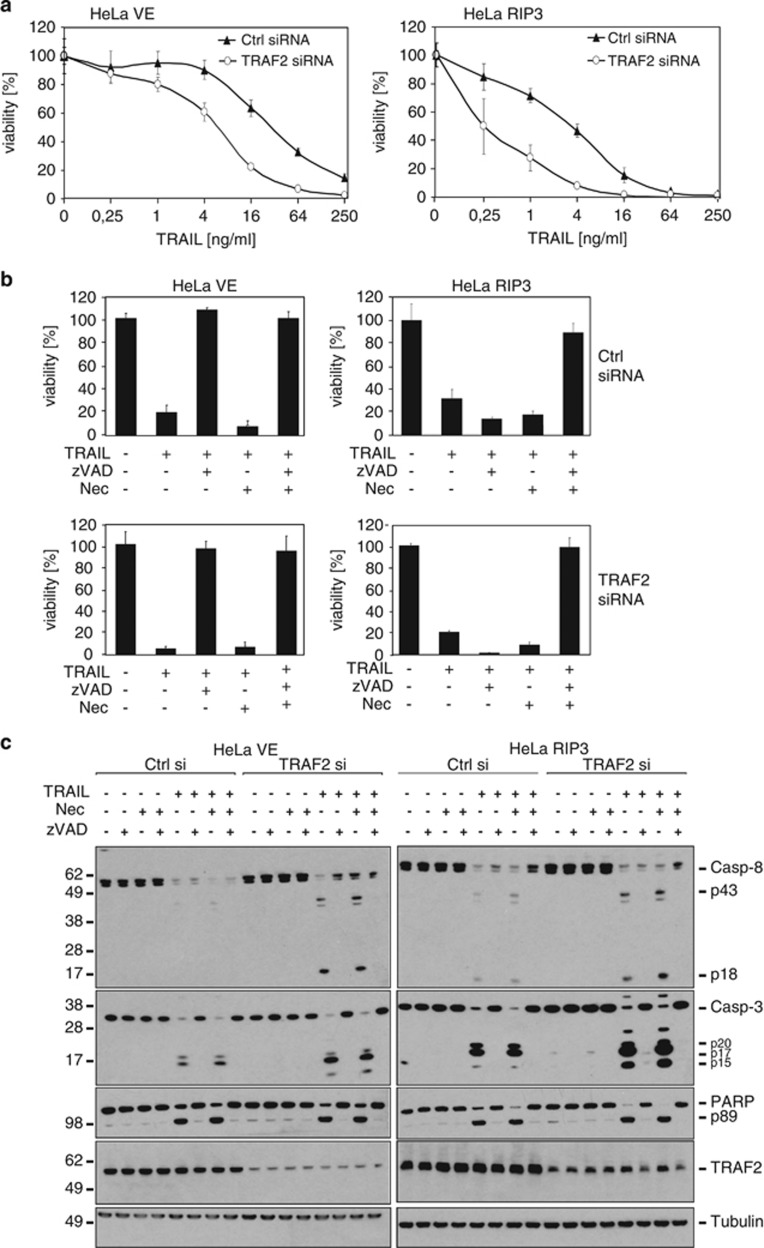
TRAIL-induced necroptosis is mediated by RIP3. HeLa cells stably infected with empty vector (VE) or a RIP3 expression plasmid were transiently transfected with TRAF2 siRNA or control siRNA (**a**). After overnight incubation with Killer-TRAIL in triplicates of the indicated concentrations, cell viability was determined by crystal violet staining. (**b**) The differently transfected HeLa cells were preincubated as indicated with zVAD-fmk (40 *μ*M) and necrostatin-1 (90 *μ*M) for 1 h and then challenged with Killer-TRAIL (64 ng/ml). Cellular viability data after overnight incubation are shown from one out of three independent experiments). (**c**) Cells were stimulated with Killer-TRAIL in the presence of the indicated mixtures of zVAD-fmk and necrostatin-1 for 3 h. Lysates were subjected to Western blot analysis (one out of two representative experiments is shown)

The data presented so far indicate that TRAF2 antagonises caspase activation and particularly necroptosis induction by TRAIL and CD95L in keratinocytes. To study whether this also applies to another epithelial cell type, we investigated the human cervical cancer cell line HeLa. As HeLa cells lack expression of RIP3,^32^ which is a crucial component of the DR-induced necroptotic pathway, we compared control-transfected HeLa cells with HeLa cells stably overexpressing RIP3 (Figure 5a, right panel). In line with the data obtained from keratinocytes, HeLa control transfectants and the RIP3-expressing transfectants were sensitised for TRAIL-induced cell death after TRAF2 KD (Figure 5a). Not unexpected in view of the lack of RIP3 expression in the HeLa control transfectants, these cells were completely rescued from TRAIL-induced cell death by zVAD-fmk even after TRAF2 KD (Figure 5b, left panel). However, in the RIP3 expressing transfectants, treatment with zVAD-fmk resulted in sensitisation for TRAIL-induced cell death. This effect was even stronger after TRAF2 KD (Figure 5b, right panel). Co-treatment of RIP3-expressing HeLa cells with a mixture of zVAD-fmk and necrostatin-1 was strongly protective irrespective of TRAF2 KD, proving the relevance of the necroptotic mode of TRAIL-induced cell death in these cells (Figure 5b). TRAIL-induced caspase processing was generally more intense in the RIP3-transfected cells and TRAF2 KD enhanced TRAIL-induced caspase activation irrespective of the RIP3 expression status (Figure 5c and Supplementary Figure S1). Altogether these data suggest (i) that RIP3 is crucial for TRAIL-induced necroptosis, (ii) that RIP3 may also enhance TRAIL-induced caspase activation and (iii) that TRAF2 antagonises both apoptosis and necroptosis induction by TRAIL.

### Depletion of cytoplasmic TRAF2/cIAP complexes by Fn14 activation and a cIAP antagonist sensitise for TRAIL-induced necroptosis

**Figure 6:**
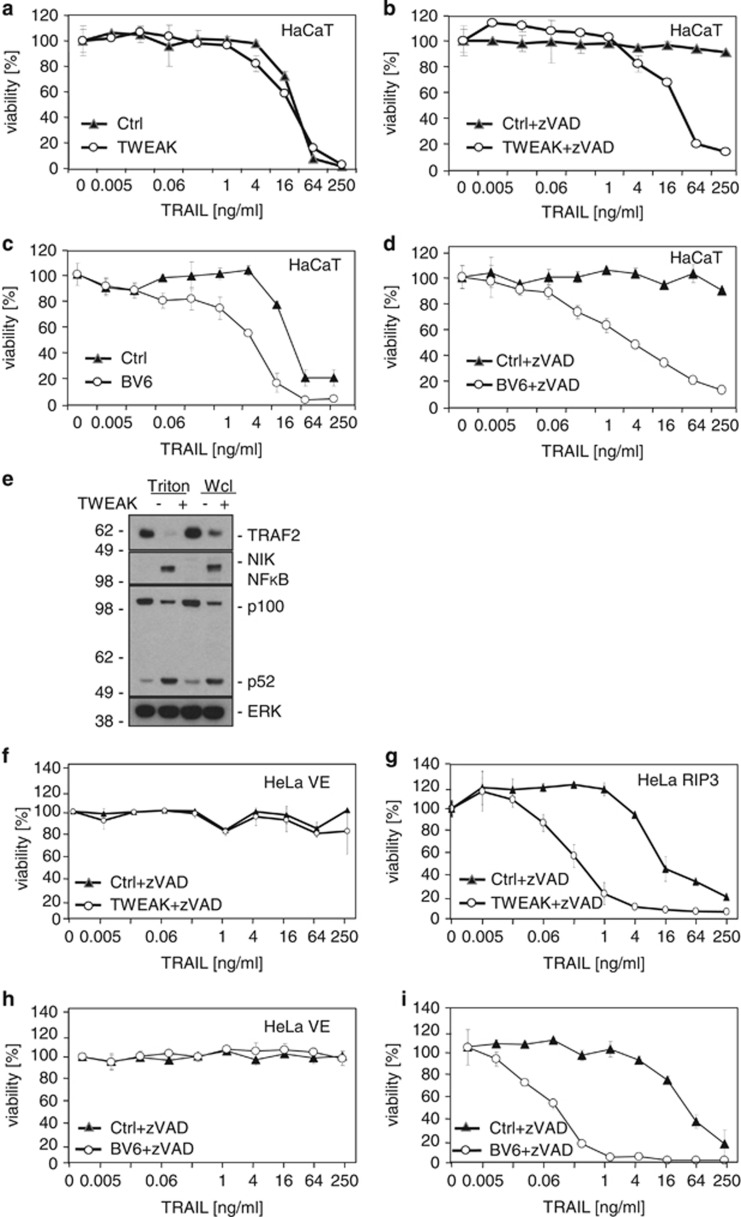
Soluble TWEAK or BV6 induce depletion of cytoplasmic TRAF2/cIAP complexes and sensitise for TRAIL-induced necroptosis. HaCaT cells were preincubated overnight with TWEAK (200 ng/ml) or medium as control (**a**, **b**) or with BV6 (10 *μ*g/ml) or diluent (**c**, **d**). The next day, cells were preincubated with zVAD-fmk (40 *μ*M) for 30 min as indicated (**b**, **d**) and stimulated with TRAIL at various concentrations for 20 h (**a**–**d**). Viability was measured by crystal violet assay as described in Materials and Methods. One representative out of four independent experiments is shown. (**e**) Cells were stimulated with TWEAK (200 ng/ml) overnight and thereafter either Triton X-100 lysates or whole cell lysates were made. Lysates were analysed for the presence of the indicated proteins by Western blotting. (**f**–**i**) Empty vector control (VE) and ectopic RIP3 expressing HeLa cells were treated as HaCaT cells in Figures 6b–d. One out of three experiments is shown

Artificial downregulation of TRAF2 expression by siRNA might have a physiological equivalent. We and others showed that stimulation of TRAF2-interacting receptors such as TNFR2 and fibroblast growth factor-inducible 14 (Fn14) lead to recruitment of TRAF2 and TRAF2-containing complexes from the cytoplasm to a Triton X-100 insoluble compartment,^33, 34, 35^ resulting in depletion of the cytoplasmic pool of TRAF2 and limitation of the availability of TRAF2 and TRAF2/cIAP complexes for other receptors. In the context of TNFR1 signalling, priming of cells with the Fn14 ligand TNF-like weak inducer of apoptosis (TWEAK) leads to a strong enhancement of TNF-induced caspase-8 activation and apoptosis.^33,34^ We thus analysed the effects of Fn14 stimulation with soluble TWEAK on TRAIL-induced cell death. In another setting we depleted intracellular cIAPs by incubating the cells with the IAP antagonist BV6.^18^ As has already been observed previously,^33^ priming with TWEAK exerted no or at best a minor sensitising effect on TRAIL-induced cell death in HaCaT cells in the absence of caspase-8 inhibitory means (Figure 6a). Of note, depletion of the Triton X-100 soluble pool of TRAF2 and, accordingly, stabilisation of NF*κ*B-inducing kinase (NIK) and processing of NF*κ*B p100 was efficient in these cells (Figure 6e). In the presence of zVAD-fmk, however, thus under necroptotic conditions, TWEAK priming resulted in a significant sensitisation for both TRAIL- and TNF-induced cell death (Figure 6b and Supplementary Figure S3). Principally similar results were obtained in cells that had been treated with the cIAP antagonist BV6 (Figures 6c and d). The necroptotic TRAIL–TWEAK crosstalk was also evident in the RIP3 expressing HeLa transfectants, but not in the parental HeLa cells lacking RIP3 expression (Figures 6f and g and Supplementary Figure S5). Again treatment with the cIAP antagonist BV6 showed similar effects as TWEAK priming (Figures 6h and i). In summary, these data show that TWEAK priming and BV6 treatment specifically sensitise for TRAIL-induced necroptosis and in this respect mimic a major effect observed with TRAF2 KD. Thus, the latter may act partly by reducing the capacity of cIAP1/2 to target RIP1.

### Sensitisation towards TRAIL-induced necroptosis in TRAF2 KD cells constitutes a genuine TRAIL signalling effect and is not an indirect effect of TNF

**Figure 7:**
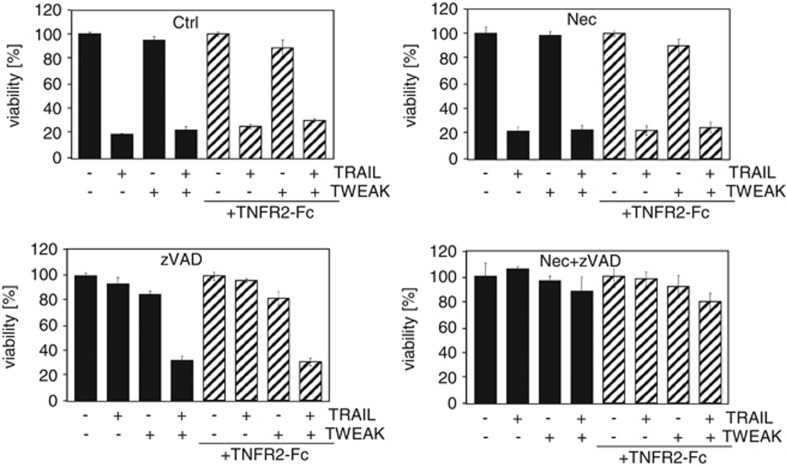
TWEAK sensitises for TRAIL-induced necroptosis independent from endogenous TNF. HaCaT cells were seeded in 96-well plates and pretreated with TWEAK (200 ng/ml) or medium control overnight. Cells were then pretreated for 1 h with zVAD-fmk (40 *μ*M), necrostatin-1 (90 *μ*M) or a combination of both and then stimulated with TRAIL (64 ng/ml) in the presence or absence of TNFR2-Fc (Enbrel) (10 *μ*g/ml). Viability was measured by crystal violet assay

Interference with the activity of the TRAF2/cIAP complex, for example, by use of second mitochondria-derived activator of caspases (SMAC) mimetics, as well as stimulation with soluble TWEAK can trigger in a cell-type dependent manner the induction of endogenous TNF and subsequent TNFR activation.^34,36^ To exclude that cytoplasmic downregulation of TRAF2 either by siRNA KD or by Fn14 signalling leads to sensitisation for necroptosis via upregulation of endogenous TNF*α* and TNFR-signalling, we performed TRAIL stimulation experiments in the presence of TNFR2-Fc (Etanercept, Enbrel), which inhibits TNF*α* and lymphotoxin *α*, or the antagonistic anti-TNF*α*-antibody Adalimumab (Humira) (Figure 7; Supplementary Figures S6 and S7). SK-OV3 cells, which are known to exert an apoptotic TWEAK response via an autocrine TNF loop served as a positive control to confirm that the TNF blockers used were effective (Supplementary Figure S2b). Effects of TRAIL or CD95L stimulation under necroptotic conditions were not altered by co-incubation with TNFR2-Fc or anti-TNF*α*, respectively (Figure 7,Supplementary Figures S2a, S6 and S7). Inhibition of classical NF*κ*B signalling, which has a major role in TNF induction but also induces survival proteins, with the I*κ*B kinase 2 (IKK2) inhibitor TPCA-1 did also not affect the cell death-enhancing effects of TRAF2 KD (Supplementary Figure S4). We thus conclude that the cell death-sensitizing properties of TRAF2 KD observed in TRAIL-stimulated cells reflect a genuine effect of TRAIL-related death signalling and not an indirect TNF effect.

## Discussion

TRAF2 is an adaptor protein that recruits cIAPs to TNFR1 and to most non-DRs of the TNF receptor family.^26^ TRAF2 is important for activation of the classical NF*κ*B pathway by these receptors but also mediates their stimulatory effects on the c-Jun N-terminal kinase pathway.^26^ TRAF2 is also involved in the constitutive cytosolic degradation of NIK, a MAP3K that triggers the noncanonical NF*κ*B signalling pathway.^37^ Most TRAF2-interacting receptors can interfere with TRAF2-dependent stimulation of NIK degradation by sequestration of TRAF2 and therefore also activate the alternative NF*κ*B pathway;^38,39^ (Figure 6e). In the context of TNFR1 signalling, TRAF2 along with TRAF1, cIAP1 and cIAP2 furthermore antagonises caspase-8 activation and inhibits apoptosis induction.^40^ Sequestration of TRAF2 and TRAF2-containing complexes by TRAF2-interacting receptors therefore also results in sensitisation for TNF-induced apoptosis.^33,34,41, 42, 43, 44, 45^

In this study, we evaluated the relevance of TRAF2 for TRAIL- and CD95L-induced cell death. TRAF2 KD indeed lowered the LC50 of TRAIL or CD95L for more than an order of magnitude in HaCaT and HeLa cells (Figures 1a, 3a and 5a). Noteworthy, TRAF2 KD not only reduced the LC50 of cell death induction by TRAIL and CD95L but also partly changed the quality of cell death. While control siRNA transfected HaCaT cells were fully rescued from TRAIL- and CD95L-induced cell death by the caspase inhibitor zVAD-fmk, TRAF2-depleted HaCaT were only partly protected and required a mixture of zVAD-fmk and the necroptosis inhibitor necrostatin-1 for survival (Figures 2b, d and 3d). HeLa cells treated with control siRNA and TRAF2 siRNA were in both cases fully protected from TRAIL-induced cell death (Figure 5b). HeLa cells, however, lack RIP3 expression and are therefore completely refractory to DR-induced necroptosis. We therefore also analysed RIP3-transfected HeLa cells with respect to the relevance of TRAF2 in TRAIL-induced cell death. In this necroptosis-competent HeLa variant, we observed roughly similar effects of TRAF2 KD on TRAIL-induced cell death as in the endogenously RIP3 expressing HaCaT cells, namely enhanced TRAIL-induced necroptosis in the presence of zVAD-fmk (Figure 5b). In sum, these data indicate that TRAF2 antagonises both DR-induced apoptosis but especially DR-stimulated necroptosis.

Initially, the survival-promoting activities of TRAF2 have mainly been investigated in the context of TNFR1 signalling and have been assigned to the ability of TRAF2 to mediate classical NF*κ*B signalling, resulting in upregulation of NF*κ*B-controlled anti-apoptotic and anti-necrotic proteins, including FLIP, ferritin heavy chain and manganese superoxide dismutase.^46, 47, 48^ However, there is increasing evidence that TRAF2 in concert with cIAP1 and cIAP2 also antagonises TNFR1-induced cell death by an NF*κ*B-independent mechanism. For example, TRAF2 depletion by simultaneous or delayed stimulation of TNFR2, in contrast to TNFR2 priming, does not inhibit TNFR1-induced NF*κ*B activation but still significantly enhances TNFR1-induced apoptosis.^44^ Indeed, the TRAF2-associated cIAPs have been found to inhibit TNF-induced RIP1-mediated activation of caspase-8 by K63 ubiquitination of RIP1.^49^ Moreover, TRAF2 has recently been identified as a K48 ubiquitin ligase for the p18 subunit of matured heterotetrameric cytosolic caspase-8.^27^ The reduction of the latter activity in TRAF2 KD cells could contribute to the sensitisation for TRAIL- and CD95L-induced caspase-dependent apoptosis observed in these cells. This cIAP1/2-independent function may also explain at least in part why TWEAK, which depletes the cytosolic pool of TRAF2-cIAP1/2 complexes, and BV6, which induces degradation of cIAP1 and cIAP2, in contrast to TRAF2 KD largely fail to enhance TRAIL-induced apoptosis although both treatments similar to the TRAF2 KD efficiently enhanced TRAIL-induced necroptosis (Figure 6). TRAF2 has been furthermore implicated in TRAIL-induced activation of the classical NF*κ*B pathway.^22,50,51^ In fact, reduced basal and/or TRAIL/CD95L-inducible expression of anti-apoptotic and anti-necrotic proteins in TRAF2 KD cells could explain both the enhancement of TRAIL/CD95L-induced apoptosis and of TRAIL/CD95L-induced necroptosis. TRAF2 KD, however, also sensitised cells in the presence of cycloheximide, an inhibitor of protein synthesis, and in the presence of the IKK2-inhibitor TPCA-1 (Supplementary Figure S4), suggesting that the pro-survival activity of TRAF2 in TRAIL- and CD95L-induced signalling is not dependent on gene induction and protein synthesis. RIP1 can promote DR-induced apoptosis but is also crucially involved in DR-induced necroptosis. As on one side, necroptotic activity of RIP1 is antagonised by cIAP1- and cIAP2-mediated ubiquitination^12^ and as on the other side cIAP1 and cIAP2 are recruited to RIP1 via TRAF2, it appears plausible that the RIP1–caspase-8 complex is the main target of the pro-survival activity of TRAF2 in context of TRAIL- and CD95L-induced cell death. Thus, the sensitising effect of TRAF2 KD on TRAIL-induced necroptosis in caspase inhibited cells is possibly caused by reduced recruitment of cIAPs to RIP1.

In view of the fact that TRAF2-interacting receptors inhibit by sequestration cytosolic TRAF2–cIAP1/2 complex-related activities, such as inhibition of the alternative NF*κ*B pathway and inhibition of TNF-induced caspase-8 activation,^40, 41, 42, 43, 44, 45^ we evaluated the effect of Fn14 stimulation on TRAIL-induced cell death. Fn14 is a broadly expressed TRAF2-interacting receptor of the TNF receptor family that, after stimulation by its ligand TWEAK, strongly triggers the alternative NF*κ*B pathway.^52,53^ Moreover, priming cells for a few hours with TWEAK results in depletion of the Triton X-100 soluble cytosolic pool of TRAF2-cIAP1/2 complexes and reduced TNF-induced TNFR1-associated ubiquitination of RIP1, which, as mentioned before, is mediated by the cIAPs.^33^ TWEAK priming thus leads to attenuated activation of the classical NF*κ*B pathway and under appropriate conditions to a tremendous sensitisation for TNF-induced cell death (200–1000-fold).^33^ In the previous cited study no major effect of TWEAK priming on TRAIL-induced apoptosis has been found.^33^ However, the effect of TWEAK on TRAIL-induced cell death had been investigated in the absence of caspase-8 inhibition and thus under conditions where DR-induced necroptosis was blocked by caspase-8.^12^ As in our current study the cell death sensitising effect of the TRAF2 KD was most prominent in the context of TRAIL- and CD95L-induced necroptosis, we looked for a possible TRAIL–TWEAK crosstalk under necroptotic conditions (caspase-8 inhibition and RIP3 expression). Indeed, under such conditions, we observed a significant enhancement of TRAIL-induced cell death by TWEAK and by preincubation with the SMAC mimetic BV6 (Figure 6). The fact that TWEAK sensitises cells for apoptosis and necroptosis in the context of TNF signalling but only for necroptosis in the context of TRAIL signalling might reflect the differential relevance of the cytosolic RIP1–caspase-8 complex for TNF- and TRAIL-induced apoptosis. In TRAIL-induced apoptosis caspase-8 is primarily activated in the receptor-associated signalling complex in a RIP1-independent manner. In the context of TNFR1-induced apoptosis, however, caspase-8 activation mainly takes place in the secondarily formed cytosolic RIP1–caspase-8 complex. This may also explain why BV6 primarily enhances TRAIL-induced necroptosis. An ability of TRAIL to induce necroptosis has often been suggested so far but only a very limited number of studies have really shown necroptosis induction by TRAIL.^14,16^ Our data confirm these observations and additionally prove in the experiments with TNFR2-Fc and with anti-TNF*α*-antibody (Figure 7,Supplementary Figures S2) that the TRAIL effect is not mediated via an autocrine TNF loop but is a direct effect of TRAIL. Most importantly, however, by showing that TRAF2 KD, TWEAK priming and BV6 treatment result in enhanced TRAIL-induced necroptosis in the presence of a caspase inhibitor, we give several lines of independent evidence for an inhibitory role of TRAF2/cIAP complexes in TRAIL- and CD95L-induced necroptotic signalling.

## Materials and Methods

### Materials

Antibodies with the following specificities were used for Western blot analysis: TRAF2, *β*-actin (abcam, Cambridge, UK), Caspase-3, cIAP1, cIAP2 (Cell signaling, Danvers, MA, USA), Caspase-8 (Enzo Life Sciences, Lausen, Switzerland), PARP, Cul3 (BD Pharmingen, Heidelberg, Germany), *α*-Tubulin (Millipore, Billerica, MA, USA) and RIP3 (Imgenex, San Diego, CA, USA). HRP-conjugated goat anti-mouse IgG1, goat anti-mouse IgG2a, goat anti-mouse IgG2b and donkey anti-rabbit antibodies were obtained from Southern Biotech (Birmingham, AL, USA) and Dianova (Hamburg, Germany). Killer-TRAIL was purchased from Alexis biosciences (Carlsbad, CA, USA). The pan-caspase-inhibitor zVAD-fmk was obtained from Bachem (Heidelberg, Germany). Necrostatin-1 was purchased from Enzo Life Sciences (Lausen, Switzerland). Fc-CD95L and Flag-TWEAK were produced as previously described.^33^ TRAF2 siRNA (HS-TRAF2_4, sequence as follows: 5′-GGACCAAGACAAGAUUGAATT-3′), alternate TRAF2 siRNA's (HS-TRAF2_5, sequence: 5′-CGAGGGCAUAUAUGAAGAATT-3′ HS TRAF2_6, sequence: 5′-GUUCGGCCUUCCCAGAUAATT-3′ HS TRAF2_7, sequence: 5′-GCUGCGGAGCAGACGUGAATT-3′) and negative control siRNA were provided by Qiagen (Hilden, Germany). siRNA transfection was performed with Lipofectamine 2000 (Invitrogen, Carlsbad, CA, USA).

### Cell culture

The spontaneously transformed keratinocyte line HaCaT was provided by P. Boukamp and cultured as previously described^31^ in Dulbecco's modified Eagle's medium supplemented with 10% foetal calf serum at 37 °C with 5% CO_2_. Primary keratinocytes were separated from neonatal foreskin as previously described,^6^ cultured in KGM (Promocell, Heidelberg, Germany) and used between passages 2–5. HeLa cells transfected with empty vector and HeLa stably transfected with RIP3 were cultured in Dulbecco's modified Eagle's medium supplemented with 10% foetal calf serum at 37 °C with 5% CO_2_. SK-OV3 cells were cultured in RPMI supplemented with 10% foetal calf serum at 37 °C with 5% CO_2_.

### siRNA transfection

For transient transfection, cells were incubated with Lipofectamine 2000 and siRNA according to the Lipofectamine transfection protocol. Briefly, 1 × 10 × 6 cells were seeded in a 10 cm culture dish. On the following day, Lipofectamine (30 *μ*l) and siRNA (20 *μ*M) were equilibrated in Opti-MEM (3 ml) and slowly added to the cells. Experiments were performed at day 1–5 post transfection. KD efficiency was confirmed by Western blot analysis and typically found between 60 and 90%. All of the experiments were performed with TRAF2_4 siRNA from Qiagen, but we could reproduce our results with other functional siRNAs for TRAF2 (Supplementary Figure S8).

### Flow cytometry

Externalisation of phosphatidylserine to the outer membrane was measured with the PE Annexin V Apoptosis Detection Kit (BD BioSciences Pharmingen, Heidelberg, Germany) according to the manufacturer's protocol. Briefly, cells were seeded into six-well plates at a density of 1 × 10 × 5/well 1 day prior to experiments. On the day of the experiment, the medium was changed and cells were stimulated with Killer-TRAIL or Fc-CD95L for the indicated times (see results). Cells were then detached, washed with ice-cold PBS and resuspended in 100 *μ*l 1 × binding buffer. As a positive control for necrosis, cells were exposed to heat-shock (55 °C for 30 min).^54^ Next, 5 *μ*l Annexin V and 5 *μ*l 7-AAD were added to each sample and cells were incubated for 15 min in the dark. Cells were measured on a FACSCanto flow cytometer (BD BioSciences, Heidelberg, Germany) and data was analysed using the FACS DIVA software (BD BioSciences).

### Western blot analysis

To prepare total cell lysates, cells were harvested, washed with PBS and directly lysed in SDS sample buffer (2% SDS, 10% glycerol, 10% *β*-mercaptoethanol, 6% 1 M TRIS pH 6.8, dH_2_O, bromophenol blue). To analyse the Triton X-100 soluble protein fraction, cells were lysed in Triton X-100 lysis buffer (1% Triton X-100, 10% glycerol, 3% 1 M TRIS pH 7.4, 6% 2 M NaCl, dH_2_O) for 30 min on ice. Thereafter, the lysates were cleared by centrifugation for 15 min at 13 000 × *g*, mixed with SDS sample buffer and boiled at 95 °C for 5 min. Lysates were separated by SDS-polyacrylamide gel electrophoresis in NuPage 4–12% Bis-Tris gels from Life technologies and transferred to nitrocellulose membranes (Amersham, GE Healthcare, Freiburg, Germany). After blocking nonspecific binding sites by incubation in Tris- or phosphate-buffered saline containing 0.1% Tween 20 and 8% dry milk, Western blot analyses were carried out with primary antibodies of the specificity of interest and horseradish peroxidase-conjugated secondary antibodies (Southern Biotech) using ECL Western blotting detection reagents (Pierce Thermo Scientific, Schwerte, Germany).

### Cytotoxicity assay

10 × 4 cells per well were seeded in 96-well plates and, after adherence, were preincubated with Flag-TWEAK overnight in the TWEAK experiments or with BV6 overnight in the BV6 experiments. The next day, cells were stimulated with the indicated doses of Fc-CD95L or Killer-TRAIL in triplicates with or without preincubation with zVAD-fmk and/or necrostatin-1. Cell viability was determined by crystal violet staining after 16–20 h.

### Data for figures

If not marked separately in the figure legends, the depicted figures show one representative experiment of at least three independent experiments performed.

## Abbreviations

TRAF2: TNF receptor-associated factor 2
TRAIL: TNF-related apoptosis inducing ligand
zVAD-fmk: Benzyloxycarbonyl-Val-Ala-Asp(OMe)-fluoromethylketone
RIP: receptor interacting protein
TWEAK: TNF-like weak inducer of apoptosis
TNF: tumor necrosis factor
DR: death receptor
FADD: Fas-associated death domain
DISC: death inducing signalling complex
cIAP: cellular inhibitor of apoptosis protein
FLIP-L: FLICE-inhibitory protein long
NFκB: nuclear factor κB
LC 50: lethal concentration 50%
siRNA: small interfering RNA
KD: knockdown
Fn14: fibroblast growth factor-inducible 14
NIK: NFκB-inducing kinase
LTα: lymphotoxin α
IKK 2: IκB kinase 2
JNK: c-Jun N-terminal kinase
MAPK: mitogen-activated protein kinase
MAP3K: MAPK kinase kinase
MnSOD: manganese superoxide dismutase
CHX: cycloheximide
SMAC: second mitochondria-derived activator of caspases
