# Supplementary material for: TRAF2 inhibits TRAIL- and CD95L-induced apoptosis and necroptosis
Source: Cell Death Dis. 2014 Oct 9;5(10):e1444–. doi: 10.1038/cddis.2014.404 (PMC4649511; doi:10.1038/cddis.2014.404)
Supplement: Supplementary Figure legends [file cddis2014404x1.doc]

**Figure legends to supplemental data**

**Figure S1: Modification of RIP proteins upon TRAIL-induced necroptosis and apoptosis in HeLa cells.** Cells were stimulated with Killer-TRAIL in the presence of the indicated mixtures of zVAD-fmk and necrostatin-1 for 3h. Whole cell lysates were subjected to Western blot analysis, detecting the indicated proteins (see also Figure 5).

**Figure S2: TRAF2 knockdown sensitizes for CD95L-induced necroptosis independent from endogenous TNF.** (a) HaCaT cells were seeded in 96-well plates in triplicates and experiments were performed basically as described for Figure 7, only that instead of using TRAIL as a death ligand, cells were stimulated with Fc-CD95L (64 ng/ml). Coincubation with 10 µg/ml TNFR2-Fc (Enbrel®) did not rescue the cells from CD95L-induced cell death. (b) As a positive control for the efficacy of the blockade of endogenous TNF by TNFR2-Fc, SK-OV3 cells were treated with soluble TWEAK, which induces TNF, with or without zVAD-fmk preincubation in the presence or absence of TNFR2-Fc as described for Figure 7.

**Figure S3: TWEAK sensitizes HaCaT keratinocytes for TNF-induced necroptosis.** Crystal violet assay was performed in HaCaT with or without TWEAK preincubation (200 ng/ml; 16 h) when incubated with different concentrations of TNFα in the presence of zVAD-fmk (40 µM) and cycloheximide (CHX; 2,5 ng/ml).

**Figure S4: TRAF2 knockdown sensitizes for TRAIL-induced necroptosis in HaCaT cells independent from NFκB signalling.** Crystal violet staining of control and TRAF2 siRNA-transfected HaCaT cells was performed basically as described in Figure 1, additionally, cells were pretreated with the IKK-inhibitor TPCA-1 (20 µM; 30 min) before adding TRAIL.

**Figure S5:** **TRAIL-induced necroptosis requires RIP3. (a)** HeLa cells stably infected with either empty vector or a RIP3 expression plasmid were seeded in 96-well plates and incubated with 200 ng/ml TWEAK or medium for 16 h. Then the cells were either pretreated with zVAD-fmk (40 µM) or necrostatin-1 (90 µM) or a combination of both or were mock-stimulated. After 1h, cells were stimulated with TRAIL (64 ng/ml). Viability was measured by crystal violet assay after 20 h. (b) Ectopic expression of RIP3 in the HeLa-RIP3 cells is proved by Western blot.

**Figure S6:** **TRAF2 knockdown sensitizes for TRAIL-induced necroptosis independent from endogenous TNF.** HaCaT cells were transiently transfected with control siRNA or TRAF2 siRNA and after 24 h seeded into 96-well plates. After 24 h, cells were either pretreated with the inhibitors zVAD-fmk (40 µM) or necrostatin-1 (90 µM) alone or in combination for 1h or directly challenged either with Killer-TRAIL (left panel) or CD95L (right panel) in the presence or absence of TNFR2-Fc (Enbrel®).

**Figure S7: TWEAK-induced sensitization for TRAIL-induced necroptosis in HaCaT cells is unchanged in the presence of the anti-TNFα antibody Adalimumab** (Humira®)**. HaCaT cells were seeded into 96-well plates and treated with TWEAK (200 ng/ml) or medium for 16 h.** Cells were then either pretreated with the inhibitors zVAD-fmk (40 µM) or necrostatin-1 (90 µM) alone or in combination for 1h or directly challenged with Killer-TRAIL (64 ng/ml) in the presence of 10 µg/ml of an irrelevant control antibody (black bars) or the anti-TNFα antibody (Humira®)

**Figure S8: Comparison of different TRAF2 siRNA’s to exclude off-target effects.** HaCaT were transfected with control – or different TRAF2- specific siRNA‘s as indicated. The next day, cells were seeded in 96-well plates and 48h after transfection were either pretreated with zVAD-fmk (40µM; 30 min) or left untreated and were then stimulated with TRAIL in the indicated doses overnight and then measured with crystal violet assay (a). Western blot was performed to control for transfection efficiency (b).
