# Supplementary material for: TRAF2 inhibits TRAIL- and CD95L-induced apoptosis and necroptosis
Source: Cell Death Dis. 2014 Oct 9;5(10):e1444–. doi: 10.1038/cddis.2014.404 (PMC4649511; doi:10.1038/cddis.2014.404)
Supplement: Supplementary Figures [file cddis2014404x2.ppt]

## Slide 1
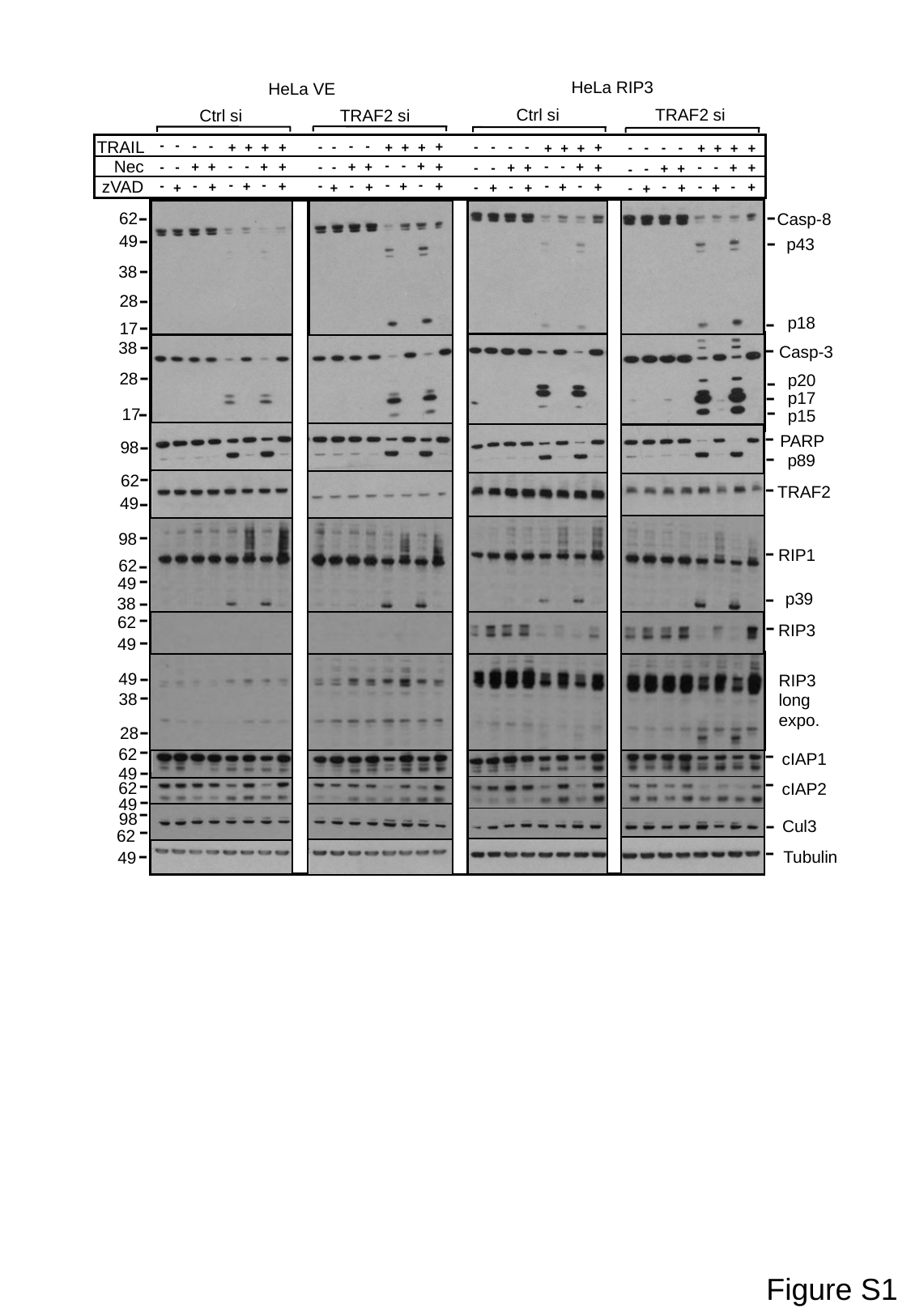

HeLa RIP3
HeLa VE
Ctrl si
TRAF2 si
Ctrl si
TRAF2 si
-
-
-
+
+
+
+
-
-
+
+
-
+
+
-
-
-
+
+
+
+
-
-
-
+
+
+
+
-
-
+
+
-
+
+
-
-
-
+
+
+
+
-
-
-
+
+
+
+
-
-
+
+
-
+
+
-
-
-
+
+
+
+
-
-
TRAIL
-
-
-
-
+
+
+
+
-
Nec
-
-
+
-
-
+
-
+
+
-
-
zVAD
-
-
-
-
-
+
+
-
+
+
-
-
-
62
Casp-8
-
-
49
p43
-
38
-
28
-
-
p18
17
-
-
38
Casp-3
-
-
28
p20
-
p17
-
-
17
p15
-
-
PARP
98
-
p89
-
-
62
TRAF2
-
49
-
98
-
RIP1
-
62
-
49
-
-
p39
38
-
-
62
RIP3
-
49
-
49
RIP3
long
expo.
-
38
-
28
-
-
62
cIAP1
-
49
-
-
62
cIAP2
-
-
49
-
98
-
Cul3
62
-
-
Tubulin
49
Figure S1

## Slide 2
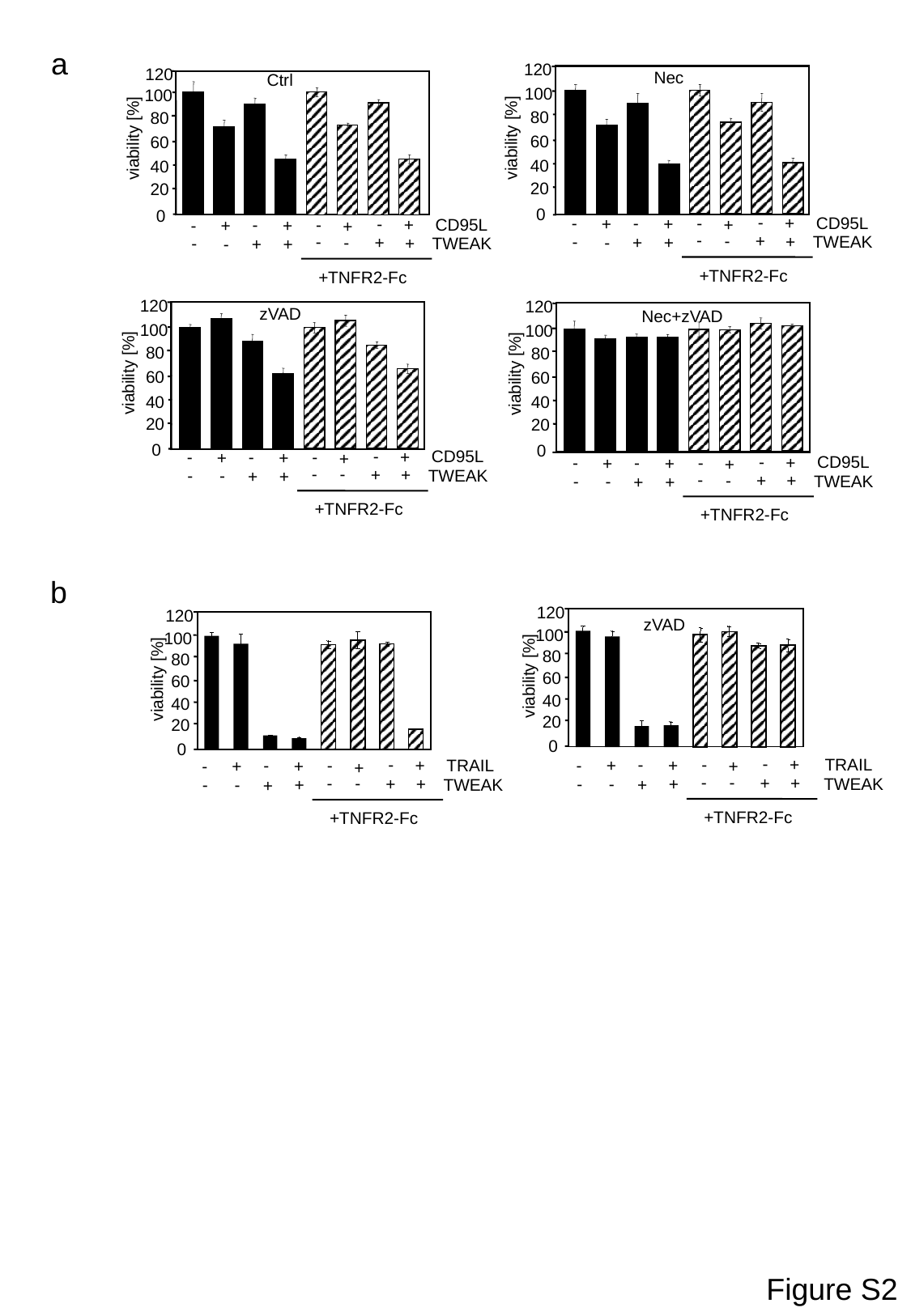

a
120
Nec
Ctrl
120
100
100
80
80
viability [%]
viability [%]
60
60
40
40
20
20
0
0
-
CD95L
-
-
+
-
+
+
+
-
-
+
+
-
TWEAK
-
+
+
+TNFR2-Fc
-
CD95L
-
-
+
-
+
+
+
-
-
+
+
-
TWEAK
-
+
+
+TNFR2-Fc
120
100
80
viability [%]
60
40
20
0
120
100
80
viability [%]
60
40
20
0
zVAD
Nec+zVAD
-
CD95L
-
-
+
-
+
+
+
-
-
+
+
-
TWEAK
-
+
+
+TNFR2-Fc
-
CD95L
-
-
+
-
+
+
+
-
-
+
+
-
TWEAK
-
+
+
+TNFR2-Fc
b
120
120
100
80
viability [%]
60
40
20
0
 zVAD
100
80
viability [%]
60
40
20
0
-
TRAIL
-
-
+
-
+
+
+
-
-
+
+
-
TWEAK
-
+
+
+TNFR2-Fc
-
TRAIL
-
-
+
-
+
+
+
-
-
+
+
-
TWEAK
-
+
+
+TNFR2-Fc
Figure S2

## Slide 3
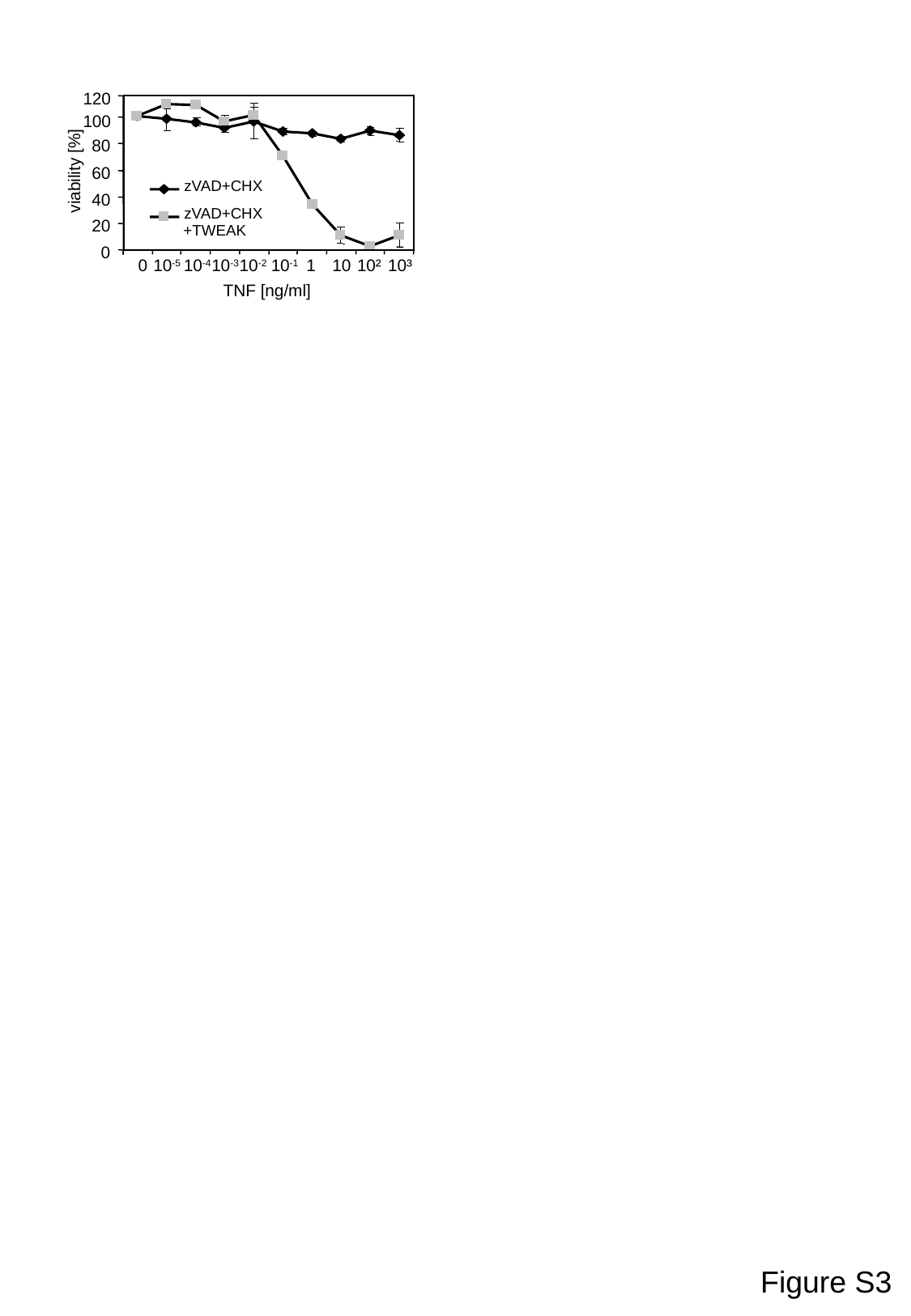

120
100
80
viability [%]
60
zVAD+CHX
40
zVAD+CHX
20
+TWEAK
0
0
10-5
10-4
10-3
10-2
10-1
1
10
10²
10³
TNF [ng/ml]
Figure S3

## Slide 4
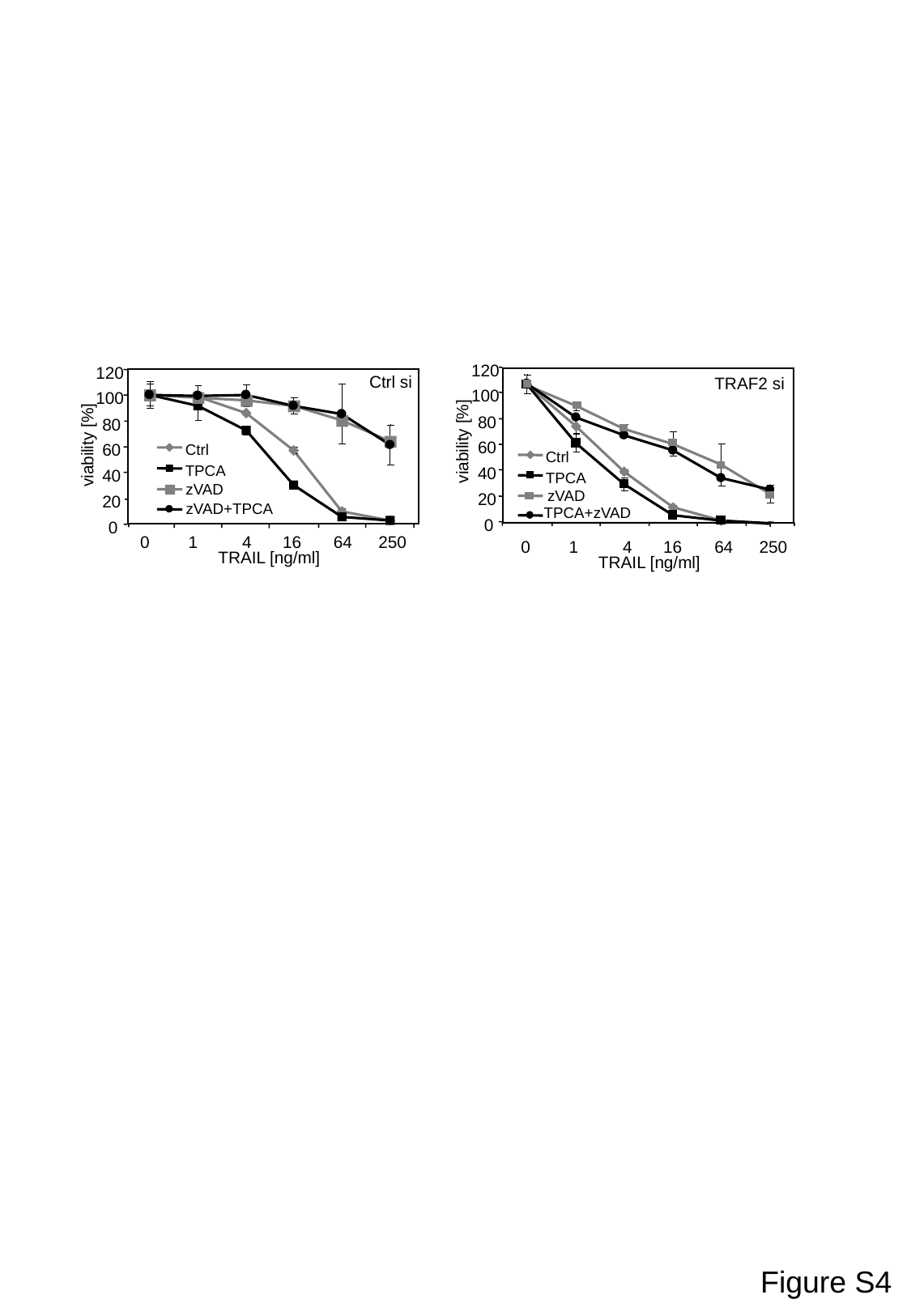

120
120
Ctrl si
TRAF2 si
100
100
80
80
viability [%]
viability [%]
60
60
Ctrl
Ctrl
TPCA
40
40
TPCA
zVAD
zVAD
20
20
zVAD+TPCA
TPCA+zVAD
0
0
0
1
4
16
64
250
0
1
4
16
64
250
TRAIL [ng/ml]
TRAIL [ng/ml]
Figure S4

## Slide 5
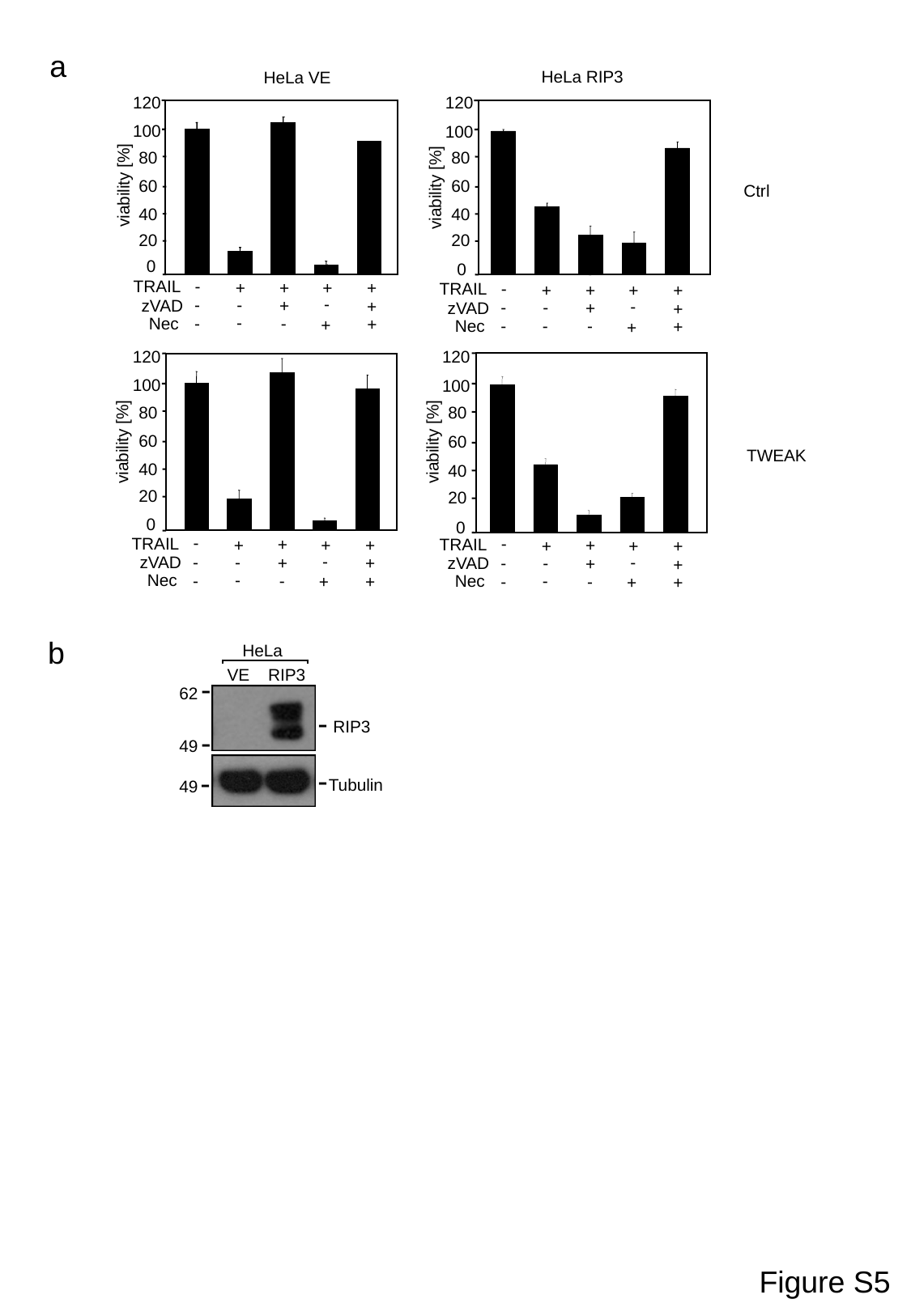

a
HeLa RIP3
HeLa VE
120
100
80
60
40
20
0
120
100
80
60
40
20
0
Ctrl
viability [%]
viability [%]
-
TRAIL
+
+
+
+
-
-
-
+
+
-
Nec
-
-
+
+
zVAD
-
TRAIL
+
+
+
+
-
-
-
+
+
-
Nec
-
-
+
+
zVAD
120
100
80
60
40
20
0
120
100
80
60
40
20
0
viability [%]
viability [%]
TWEAK
-
TRAIL
+
+
+
+
-
-
-
+
+
-
Nec
-
-
+
+
zVAD
-
TRAIL
+
+
+
+
-
-
-
+
+
-
Nec
-
-
+
+
zVAD
b
HeLa
VE RIP3
-
62
-
 RIP3
-
49
-
-
Tubulin
49
Figure S5

## Slide 6
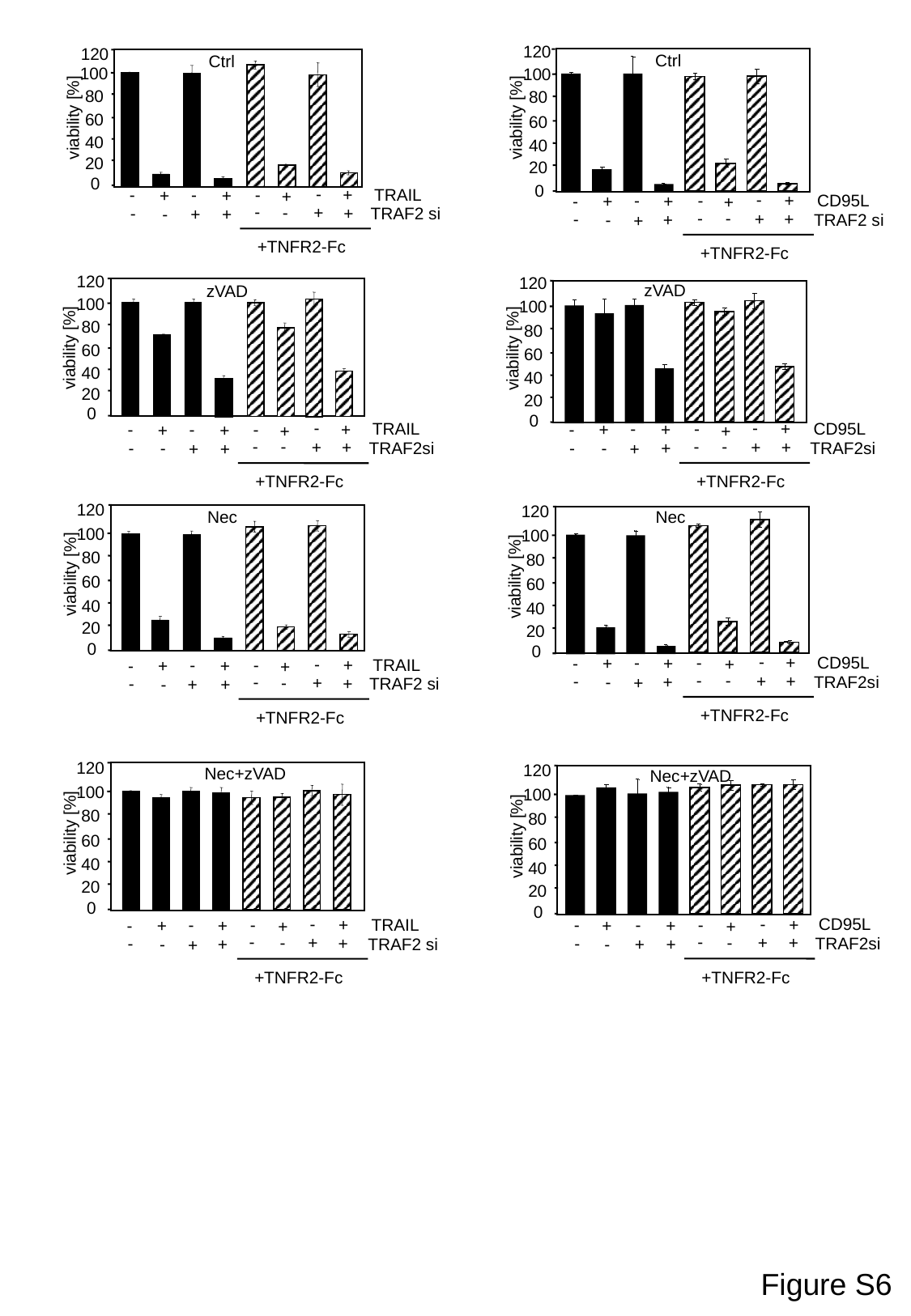

120
120
100
80
60
40
20
0
viability [%]
Ctrl
Ctrl
100
80
viability [%]
60
40
20
-
TRAIL
-
-
+
-
+
+
+
-
-
+
+
-
TRAF2 si
-
+
+
+TNFR2-Fc
0
-
CD95L
-
-
+
-
+
+
+
-
-
+
+
-
TRAF2 si
-
+
+
+TNFR2-Fc
120
100
80
60
40
20
0
viability [%]
120
100
80
60
40
20
0
viability [%]
zVAD
zVAD
-
CD95L
-
-
+
-
+
+
+
-
-
+
+
-
TRAF2si
-
+
+
+TNFR2-Fc
-
TRAIL
-
-
+
-
+
+
+
-
-
+
+
-
TRAF2si
-
+
+
+TNFR2-Fc
120
100
80
60
40
20
0
viability [%]
Nec
Nec
120
100
80
60
40
20
0
viability [%]
-
CD95L
-
-
+
-
+
+
+
-
-
+
+
-
TRAF2si
-
+
+
+TNFR2-Fc
-
TRAIL
-
-
+
-
+
+
+
-
-
+
+
-
TRAF2 si
-
+
+
+TNFR2-Fc
 Nec+zVAD
 Nec+zVAD
120
100
80
60
40
20
0
viability [%]
120
100
80
60
40
20
0
viability [%]
-
CD95L
-
-
+
-
+
+
+
-
-
+
+
-
TRAF2si
-
+
+
+TNFR2-Fc
-
TRAIL
-
-
+
-
+
+
+
-
-
+
+
-
TRAF2 si
-
+
+
+TNFR2-Fc
Figure S6

## Slide 7
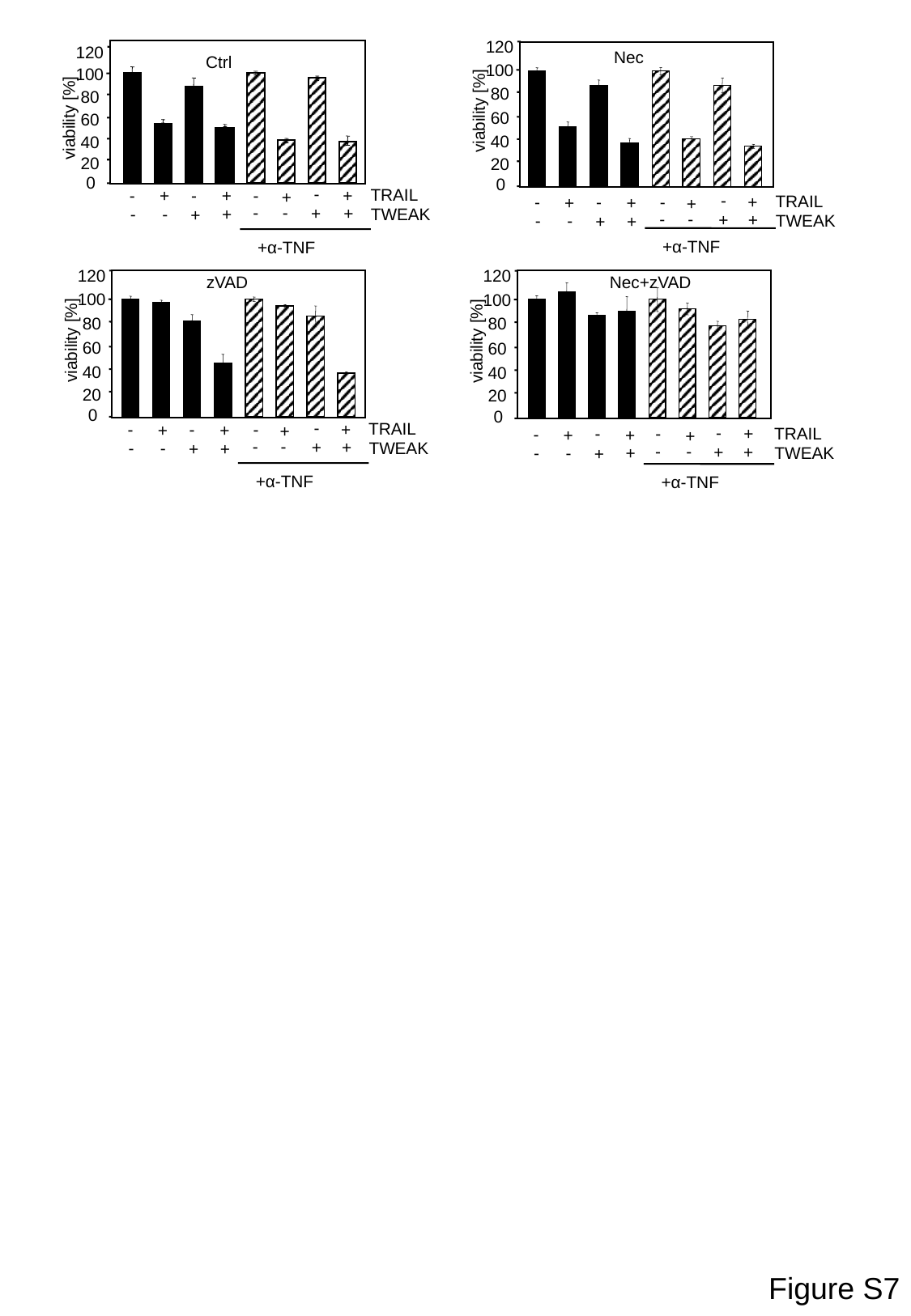

120
100
80
60
40
20
0
viability [%]
Nec
120
100
80
60
40
20
0
viability [%]
Ctrl
-
TRAIL
-
-
+
-
+
+
+
-
-
+
+
-
TWEAK
-
+
+
+α-TNF
-
TRAIL
-
-
+
-
+
+
+
-
-
+
+
-
TWEAK
-
+
+
+α-TNF
 Nec+zVAD
120
100
80
60
40
20
0
viability [%]
120
100
80
60
40
20
0
viability [%]
zVAD
-
TRAIL
-
-
+
-
+
+
+
-
-
+
+
-
TWEAK
-
+
+
+α-TNF
-
TRAIL
-
-
+
-
+
+
+
-
-
+
+
-
TWEAK
-
+
+
+α-TNF
Figure S7

## Slide 8
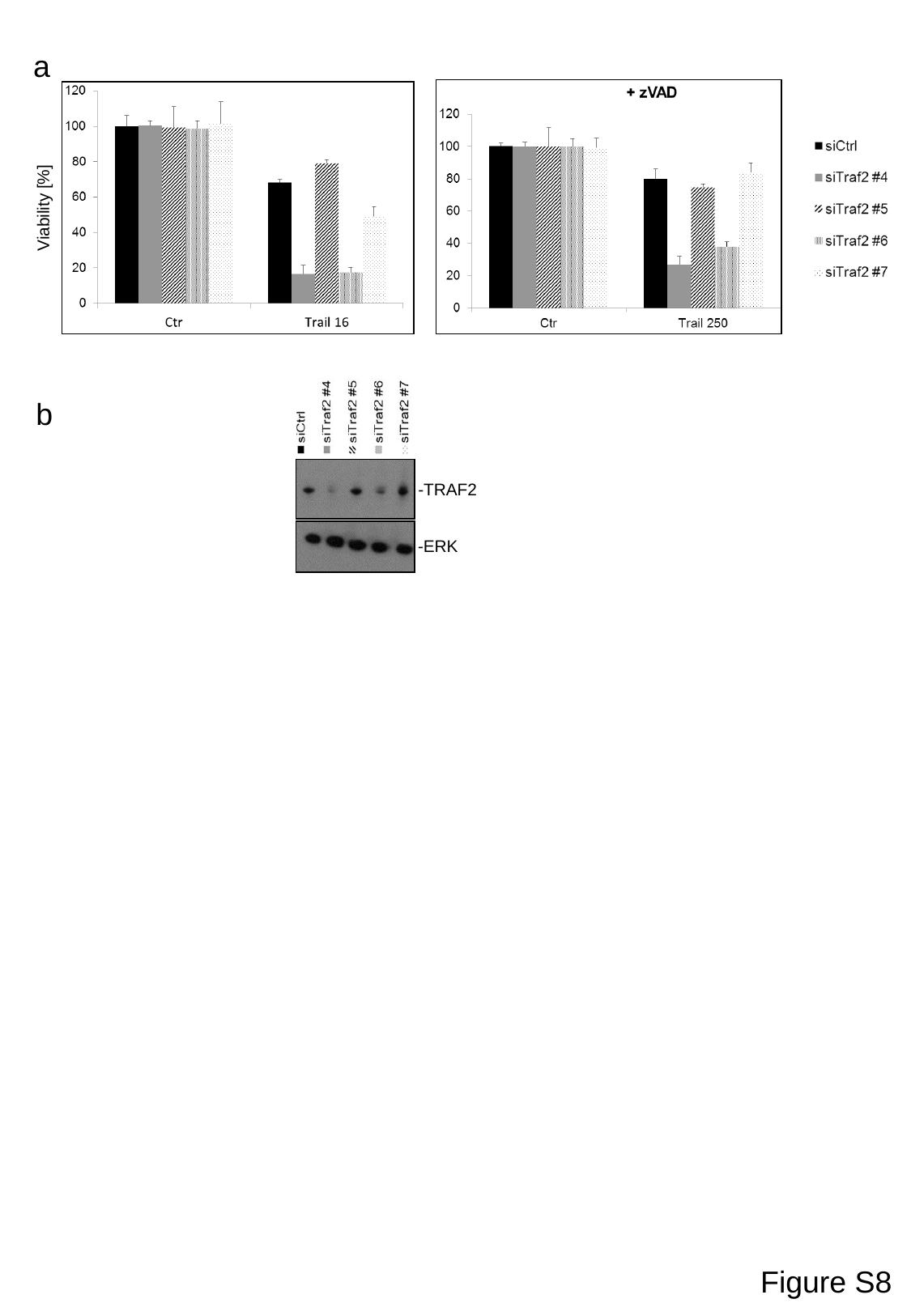

a
Viability [%]
b
-TRAF2
-ERK
Figure S8
